# THE ROLE OF IMPROVED SOCIAL SUPPORT FOR HEALTHY EATING IN A LIFESTYLE INTERVENTION: TEXERCISE SELECT

**DOI:** 10.1093/geroni/igz038.2454

**Published:** 2019-11-08

**Authors:** Aya Yoshikawa, Matthew L Smith, Shinduk Lee, Samuel D Towne Jr., Marcia G Ory

**Affiliations:** 1 Center for Population Health and Aging, Texas A&M University, College Station, Texas, United States; 2 Health Management & Informatics, University of Central Florida, Orlando, Florida, United States; 3 Texas A&M Center for Population Health and Aging, College Station, Texas, United States

## Abstract

Healthy diet is essential to the management of chronic conditions such as cardiovascular disease and diabetes. Research suggests an association between social support and dietary behavior, yet the relationship is not fully explored. The role of social support in nutrition consumption was examined among older participants in a group-based lifestyle enhancement program (Texercise Select) designed to improve dietary behaviors and physical activity and related supports. Factor analysis and structural equation modeling were performed using secondary data from a quasi-experimental study of participants who completed a baseline survey and three-month follow-up (Intervention group N = 211, comparison group N = 175). The majority of participants were age 70 years or older (Mean = 74.30, SD = 8.54), female (82.1%), and had at least two chronic conditions (63.5%). The two groups did not differ in baseline levels of nutrition intake or social support. Program participants improved in terms of intake of water and fruits/vegetables as well as social support. Structural equation models adjusting for the effect of baseline scores indicated that the intervention effect on fruits/vegetable intake (β = 0.19, p < 0.001) was partially mediated (β = 0.03, p = 0.021) by social support for planning and keeping dietary goals and reducing barriers to healthy eating (X2/df = 1.89; RMSEA = 0.04; CFI = 0.99; TLI = 0.99; SRMR=0.02). Findings suggest that programs designed to enhance social support may be effective in improving dietary behaviors among older adults. Future research should investigate various types of social support for promoting healthy diets.

